# Ribosome Homeostasis Regulated by SETD2 Preserves Intestinal Epithelial Barrier

**DOI:** 10.1002/advs.202508168

**Published:** 2026-01-04

**Authors:** Hanyu Rao, Aiting Wang, Yue Xu, Wenxin Feng, Chunxiao Ma, Ziyi Wang, Wei Zhang, Wenqiong Su, Xiuying Xiao, Wei‐Qiang Gao, Xianting Ding, Li Li

**Affiliations:** ^1^ State Key Laboratory of Systems Medicine for Cancer Shanghai Cancer Institute School of Medicine and School of Biomedical Engineering Ren Ji Hospital Shanghai Jiao Tong University Shanghai China; ^2^ State Key Laboratory of Systems Medicine for Cancer Institute for Personalized Medicine and Med‐X Research Institute Shanghai Jiao Tong University Shanghai China; ^3^ Department of Anesthesiology and Surgical Intensive Care Unit School of Medicine and School of Biomedical Engineering Xinhua Hospital Shanghai Jiao Tong University Shanghai China; ^4^ Department of Oncology School of Medicine Ren Ji Hospital Shanghai Jiao Tong University Shanghai China

**Keywords:** intestinal barrier, ribosome biogenesis factors (RBFs), ribosome homeostasis, ribosomal proteins (RPs), SET domain–containing 2 (SETD2)

## Abstract

Strict regulation of epithelial cells is crucial for maintaining intestinal barrier integrity and preventing intestinal diseases. While transcriptional regulation is well recognized as vital in this process, translational regulation is equally important. SETD2, a methyltransferase, is involved in transcriptional regulation to maintain intestinal epithelial barrier function. However, its role in translation remains largely unexplored. Here, we found SETD2 deficiency leads to the downregulation of ribosome biogenesis progress coupled with transcriptome‐proteome discordance. Further ribosome profiling sequencing analyses showed reduced translational efficiency of cell adhesion and junction signatures in impaired intestinal epithelial barrier. Mechanistically, SETD2 ablation causes dysregulation and recruitment disorders of ribosome biogenesis factors, impairing the composition and distribution of ribosomal proteins. This disruption of ribosome biogenesis and homeostasis results in translational disorder of barrier maintenance genes, thereby compromising the intestinal barrier. Collectively, our findings unveil a previously unappreciated role of ribosome biogenesis and translational regulation in safeguarding intestinal epithelial barrier, and disclose a previously undiscovered role of SETD2 in modulating ribosome homeostasis.

## Graphical abstract

SETD2 ablation causes dysregulation and recruitment defects of ribosome biogenesis factors, resulting in translational disorders of barrier maintenance genes, thereby compromising the intestinal barrier. These findings unveil a previously unappreciated role of ribosome biogenesis and translational regulation in preserving the intestinal epithelial barrier, and disclose a previously undiscovered role of SETD2 in modulating ribosome homeostasis.

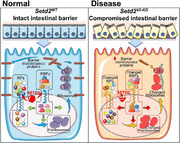

## 1 Introduction

The intestinal barrier, composed of intestinal epithelial cells (IECs), is essential for maintaining intestinal homeostasis by shielding host tissues from infections, environmental toxins, pollutants, and allergens [1]. Dysfunctional IECs contribute to the compromised intestinal epithelial barrier, a prominent feature of numerous diseases, including inflammatory bowel disease (IBD) [2, 3] and colorectal cancer (CRC) [4]. Notably, IECs proliferate rapidly and renew frequently, making strict regulation of cell adhesion and junction of IECs crucial to maintaining intestinal barrier integrity [5]. However, the underlying molecular mechanisms governing epithelial barrier maintenance  remain largely ambiguous.

Translation of mRNA, one of the most energetically demanding processes within a cell, is meticulously regulated to meet cellular demands. mRNA levels within a cell do not necessarily correlate with protein abundance [6], underscoring the high variability in translational efficiency [7]. Dysregulated translation has been linked to numerous human diseases [8], including immunodeficiency [9], metabolic disorders,^[^ ^10^ ^]^ neurological disorders [11], and cancer [12]. Ribosomes, multi‐unit complexes responsible for translation, are generated via ribosome biogenesis, which is critical for protein production and plays essential roles in cell proliferation, differentiation, apoptosis, senescence, and transformation [13, 14, 15, 16]. Dysregulation of ribosome is increasingly recognized as a critical factor in the pathogenesis of various human diseases [17]. Particularly, aberrant ribosome function and impaired  translation are known to be crucial for intestinal stem cell identity [18] and gastrointestinal cancers [19, 20, 21]. However, the specific contributions of ribosome homeostasis and translational regulation to intestinal epithelial barriers have not yet been definitively established.

Mutations in the methyltransferase SET‐domain‐containing 2 (SETD2) are common in colitis and colorectal cancer samples characterized by impaired epithelial barrier function [22, 23]. As a multifunctional protein, SETD2 has been shown to participate in diverse biological processes including transcriptional regulation, chromosome segregation, DNA damage repair, and alternative splicing [24, 25]. Our previous studies demonstrated that SETD2 plays a role in mitigating colitis [26] and inhibiting colorectal cancer progression [27] underscoring its positive function in maintaining the functional intestinal epithelial barrier. While the role of SETD2 in transcriptional regulation is well established, its implication in translation remains largely undefined in intestinal barrier maintenance.

In this study, we conduct systematic multi‐omics characterizations on Setd2^WT^ and Setd2^Vil‐KO^ mice challenged with dextran sulfate sodium (DSS), an inducer of intestinal damage, to investigate the function of SETD2 in safeguarding functional intestinal barrier. Specifically, we uncover discordant proteomic and transcriptomic signatures, particularly among genes related to cell adhesion and junction, following SETD2 deficiency. Ribosomal analyses demonstrated that SETD2 ablation‐induced dysregulation of ribosome biogenesis results in disrupted ribosome homeostasis and translational disorder on cell adhesion and junction genes. Therefore, our findings uncover the function of SETD2 in modulating ribosome homeostasis and unveil a previously unappreciated role of ribosome biogenesis and translational regulation in preserving intestinal epithelial barrier integrity.

## 2 Results

### 2.1 Ribosome Biogenesis Factors are Dysregulated Following SETD2 Ablation in DSS‐Induced Intestinal Injury Mice Model

As outlined in our prior study [26] and Figure S1A,B, we identified frequent SETD2 mutations in colorectal cancers, observed reduced SETD2 levels in clinical specimens from IBD and CRC patients, and found that SETD2 deficiency leads to impaired intestinal barrier integrity. These results demonstrate a critical role of SETD2 in preserving intestinal barriers and inhibiting intestinal diseases.

**FIGURE 1:**
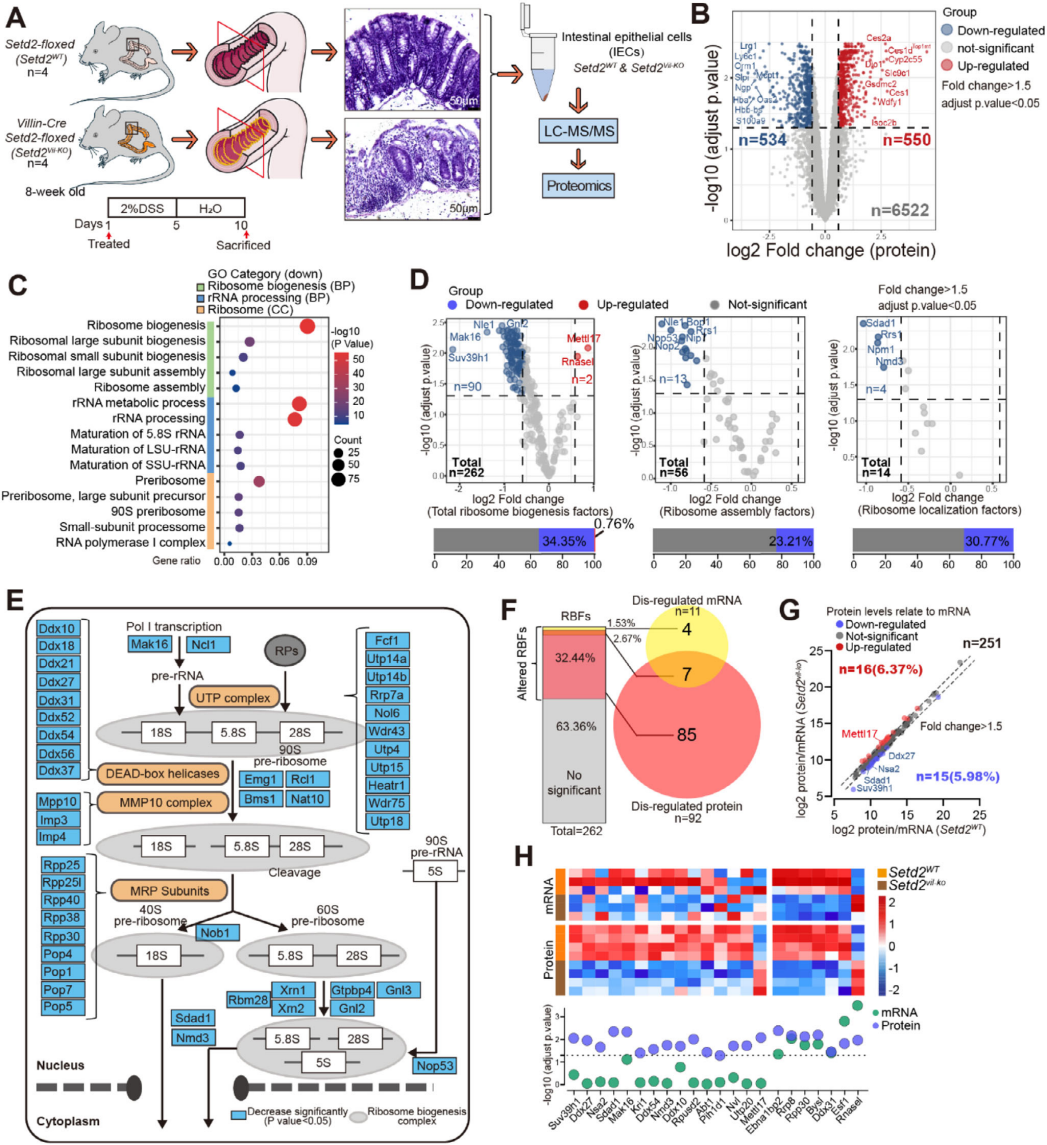
Ribosome biogenesis factors are dysregulated following SETD2 deletion. **(A)** Schematic representation of the DSS protocol used to induce acute intestinal damage. **(B)** Volcano plot of protein alterations in IECs following Setd2 deletion. **(C)** Significantly altered GO pathways related to ribosome biogenesis between IECs from DSS‐treated Setd2^Vil‐KO^ and Setd2^WT^ mice based on differential proteins. **(D)** Volcano plot of RBFs alterations following Setd2 deletion. **(E)** Schematic representation of disordered ribosome biogenesis pathway in IECs following Setd2 deletion. The significantly decreased RBFs were highlighted in blue (P. value<0.05 and fold change>1.5). **(F)** Venn diagram showing the number of RBFs displaying expression changes on mRNA and protein levels in IECs following *Setd2* deletion. **(G)** Differential expression between proteins and mRNA levels of RBFs in IECs following *Setd2* deletion. Up‐ and down‐regulated RBFs compared with their mRNA levels are shown in red and blue, respectively. **(H)** Heat map and quantitative analysis of mRNA (n = 3 per genotype) and protein (n = 4 per genotype) levels of RBFs between IECs from DSS‐treated Setd2^Vil‐KO^ and Setd2^WT^ mice. Statistical analyses were performed with a two‐tailed Student's t‐test.

To further assess the impact of SETD2 loss in intestinal epithelium, we employed an intestinal epithelium‐specific Setd2 knockout mouse strain (Villin‐Cre; Setd2^flox/flox^ mice, hereinafter referred to as Setd2^Vil‐KO^ mice) and a control mouse strain (hereinafter referred to as Setd2^WT^ mice). For statistical analysis, mice of each strain were challenged with 2% DSS for 5 days, followed by 5 days of regular drinking water, to evaluate their susceptibility to damage (Figure 1A). Upon DSS administration, Setd2^Vil‐KO^ mice exhibited greater body weight loss, more widespread intestinal tissue disruption, and increased intestinal permeability compared with Setd2^WT^ mice, indicating aggravated intestinal damage (Figure 1A; Figure S1C–E). Subsequently, intestinal epithelial cells (IECs) from Setd2^WT^ and Setd2^Vil‐KO^ mice were subjected to LC‐MS/MS proteomics analysis to delineate the regulatory mechanisms underlying epithelial response to injury following SETD2 ablation. Proteomics analysis identified 1,084 differentially expressed proteins (adjusted p‐value < 0.05, fold change > 1.5), with 534 downregulated proteins (49.26%) and 550 upregulated proteins (50.74%) in IECs from Setd2^Vil‐KO^ mice compared with Setd2^WT^ mice (Figure 1B; Figure S2A).

Notably, Gene Ontology (GO) analysis revealed marked enrichment of downregulated ribosome biogenesis‐associated proteins upon SETD2 ablation (Figure 1C; Figure S2B). At the gene level, among the 262 ribosome biogenesis factors (RBFs) analyzed, 92 (34.35%) were altered, with a majority being downregulated. Specifically, 23.21% of ribosome assembly factors and 30.77% of ribosome localization factors were downregulated (Figure 1D). These altered RBFs encompass nearly every key step of ribosome synthesis, signifying substantial and widespread changes in the process of ribosome biogenesis (Figure 1E). However, by integrating our transcriptomics data (GSE 151968), we found that only 7 out of 92 RBFs exhibited concurrent regulation at both mRNA and protein levels. The majority (87 out of 92) only showed altered protein levels following SETD2 ablation (Figure 1F), suggesting a potential protein‐level regulatory mechanism affecting RBFs. At the gene level, 6.37% (n = 16) and 5.98% (n = 15) of total RBFs were upregulated and downregulated at the protein level compared with their mRNA levels, respectively (Figure 1G). The most significantly changed RBFs were shown in Figure 1H and Figure S2C,D. To delineate direct SETD2‐specific effects from inflammation‐associated outcomes, we conducted LC‐MS/MS proteomic analysis on IECs from untreated Setd2^WT^ and Setd2^Vil‐KO^ mice, identifying 393 differentially expressed proteins (adjusted p‐value < 0.05, fold change > 1.5) (Figure S2E,F). GO analysis further revealed dysregulated ribosome biogenesis process in SETD2‐deficient IECs, consistent with our observations in DSS‐treated mice (Figure S2G). Most altered proteins (196 out of 220) exhibited consistent expression trends upon *Setd2* deletion. Notably, all 15 RBFs were downregulated under both conditions (Figure S2H). These results demonstrated that RBFs are dysregulated following SETD2 deficiency, suggesting a potential regulatory role of SETD2 on ribosome biogenesis in IECs.

### 2.2 SETD2 Deficiency Leads to Discordant Transcriptional and Translational Signatures in IECs

**FIGURE 2:**
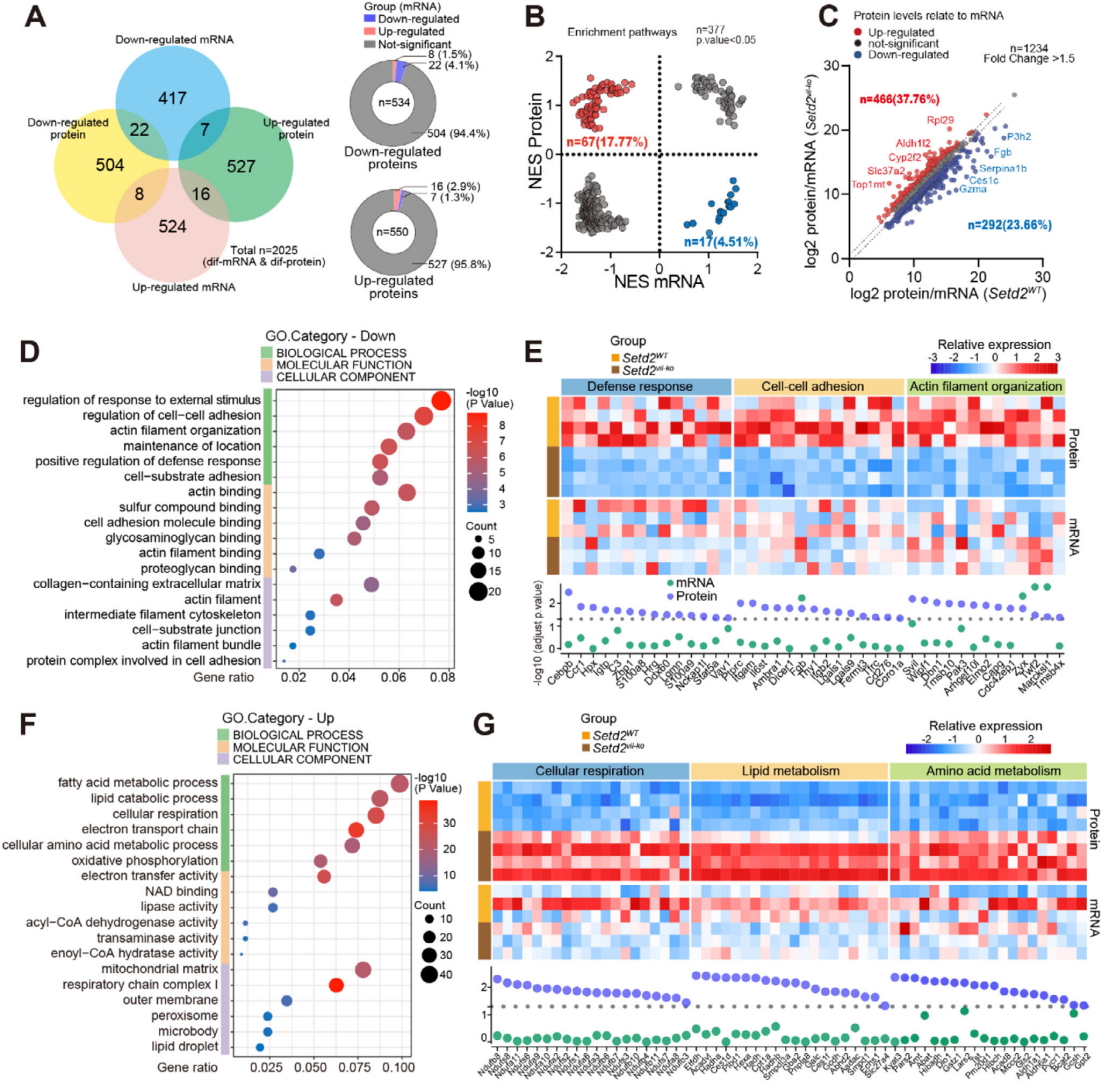
SETD2 deficiency leads to discordant proteomic and transcriptomic signatures. **(A)** Venn diagram showing the number of genes displaying expression changes on mRNA and protein levels. **(B)** GSEA analysis of altered pathways following Setd2 deletion at mRNA (x‐axis) and protein (y‐axis) levels based on GO subset in AmiGO database. Normalized enrichment scores (NES) of GO terms are plotted. **(C)** Differential expression between proteins and mRNA levels in IECs following Setd2 deletion. Up‐ and down‐regulated proteins compared with their mRNA levels are shown in red and blue, respectively. **(D and F)** Significantly altered GO pathways following Setd2 deletion based on discordant genes. **(E and G)** Heat map and quantitative analysis of discordant genes (mRNA, n = 3; protein, n = 4; per genotype) between Setd2^Vil‐KO^ and Setd2^WT^ IECs. Statistical analyses were performed with a two‐tailed Student's t‐test.

Given the essential role of ribosome biogenesis in translational control, we further conducted integrative multi‐omics analysis of transcriptomics and proteomics to systematically characterize post‐transcriptional dysregulation following SETD2 depletion (Figure S3A,B). Notably, only 4.1% of down‐regulated proteins and 1.3% of up‐regulated proteins showed corresponding changes on mRNA level, respectively. The majority showed discordant protein and mRNA levels following SETD2 ablation (Figure 2A). Utilizing GO analyses and gene set enrichment analyses (GSEA), we observed inconsistency in 22.28% of pathways between mRNA and protein levels (Figure 2B; Figure S3C,D). At the gene level, 37.76% (n = 466) and 23.66% (n = 293) of total differential genes were upregulated and downregulated at the protein level compared with their mRNA levels following SETD2 ablation, respectively (Figure 2C). These results highlight the marked discrepancy between mRNA and protein expression patterns following SETD2 ablation in IECs. Notably, GO enrichment analysis of proteomic datasets revealed that protein stability and degradation processes remained unaltered upon SETD2 ablation in both DSS‐treated and untreated mice (Figure S3E), supporting a regulatory role of SETD2 in protein translation.

Subsequently, we conducted an in‐depth examination of discordant genes to elucidate the divergent proteomic and transcriptomic profiles that emerged after SETD2 ablation. GO enrichment analysis revealed a notable downregulation of genes associated with defense response, cell adhesion, and actin cytoskeleton reorganization pathways (Figure 2D,E). Simultaneously, there was a significant upregulation of genes involved in cellular respiration, lipid metabolism, and amino acid metabolism pathways (Figure 2F,G). These findings collectively indicate substantial alterations in cellular location maintenance and metabolic processes of IECs following SETD2 ablation.

### 2.3 SETD2 Loss‐Induced Translational Disorder Affects Translation of Barrier Maintenance Proteins

**FIGURE 3:**
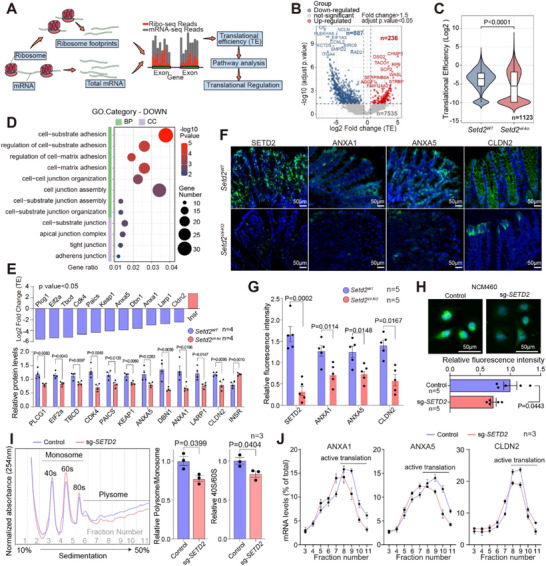
SETD2 deficient IECs exhibit a disordered translational landscape on cell adhesion and junction signature. **(A)** Schematic representation of the Ribo‐seq analyses of IECs from DSS‐treated Setd2^Vil‐KO^ and Setd2^WT^ mice. **(B)** Volcano plot of TE alterations in IECs following Setd2 deletion. **(C)** Overall TE of differentially translational genes. **(D)** Significantly altered GO pathways based on genes with downregulated TE in IECs from DSS‐treated Setd2^Vil‐KO^ mice versus Setd2^WT^ mice. **(E)** Log‐transformed change levels for TE of cell junction genes, and their relative protein levels between IECs from DSS‐treated Setd2^Vil‐KO^ (n = 4) and Setd2^WT^ (n = 4) mice. **(F‐G)** Representative images and metrics showing protein expression in colon sections from DSS‐treated *Setd2^Vil‐KO^* and Setd2^WT^ mice. **(H)** Representative images and metrics showing translational output in SETD2 wildtype and knockout NCM460 cells. Scale Bars: 50 µm. **(I)** Representative polysome profiling results of NCM460 cells with or without *SETD2* knockout. (**J**) RT‐qPCR analyses on fractions from polysome profiling. Statistical analyses were performed with a two‐tailed Student's t‐test. Data are represented as mean ± SEM.

Since RBFs are critical to functional ribosome and translation process [13], we further utilized ribosome profiling sequencing (Ribo‐seq) to investigate genome‐wide translational efficiency (TE) changes following SETD2‐deficiency‐induced RBFs and post‐transcriptional disorder. In this process, mRNA undergoing translation, represented as ribosome‐protected fragments (RPFs), was sequenced and compared with total mRNA sequencing data to illustrate changes in the TE of genes (Figure 3A). The high quality of our Ribo‐seq libraries is evidenced by several key observations, confirming their suitability for downstream analysis (Figure S4A–D). At the gene level, a total of 1123 differentially translated genes (adjusted p‐value < 0.05, fold change > 1.5) were identified, with a majority being downregulated (n = 887, 79.33%) following SETD2 ablation in IECs (Figure 3B). Notably, this specific set of differentially translated genes also exhibited a reduction in overall TE (Figure 3C). Further GO and KEGG enrichment analysis revealed distinct alterations in cell adhesion and junction pathways, which were significantly downregulated in IECs from Setd2^Vil‐KO^ mice compared with Setd2^WT^ mice (Figure 3D; Figure S4E), aligning with the multi‐omics data presented in Figure 2. The downregulation of cell adhesion and junction processes was further substantiated by the decreased expression of cell adhesion and junction proteins (adjusted p‐value < 0.05) (Figure 3E). Specifically, proteins such as ANXA1, ANXA5, and CLDN2 exhibited significant reductions at the protein level following SETD2 ablation in IECs (Figure 3F,G), yet their corresponding mRNA levels remained unchanged (Figure S4F).

To further elucidate translational defects following SETD2 ablation, we performed O‐propargyl‐puromycin (OPP) labeling and polysome profiling on the normal human IECs (NCM460) with or without *SETD2* knockout to measure changes in protein synthesis. These assays revealed decreased translational output (Figure 3H) and a reduction in the pool of polysomes (Figure 3I) upon SETD2 deficiency, collectively indicating a diminished translational activity. Reduced 40S/60S ratio and decreased pre‐rRNA production further indicated an altered ribosome homeostasis caused by SETD2 ablation (Figure S4G). In line with our observation in mice, RT‐qPCR analyses on fractions from polysome profiling confirmed distinct alterations in TE of *ANXA1, ANXA5*, and *CLDN2* genes (Figure 3J). These findings highlight that the TE of cell adhesion and junction genes is downregulated following SETD2 ablation, which accounts for the decreased abundance of these genes' protein products in SETD2 deficient IECs.

### 2.4 SETD2 Ablation Causes a Recruitment Disorder of Ribosome Biogenesis Factors and Ribosomal Proteins

**FIGURE 4:**
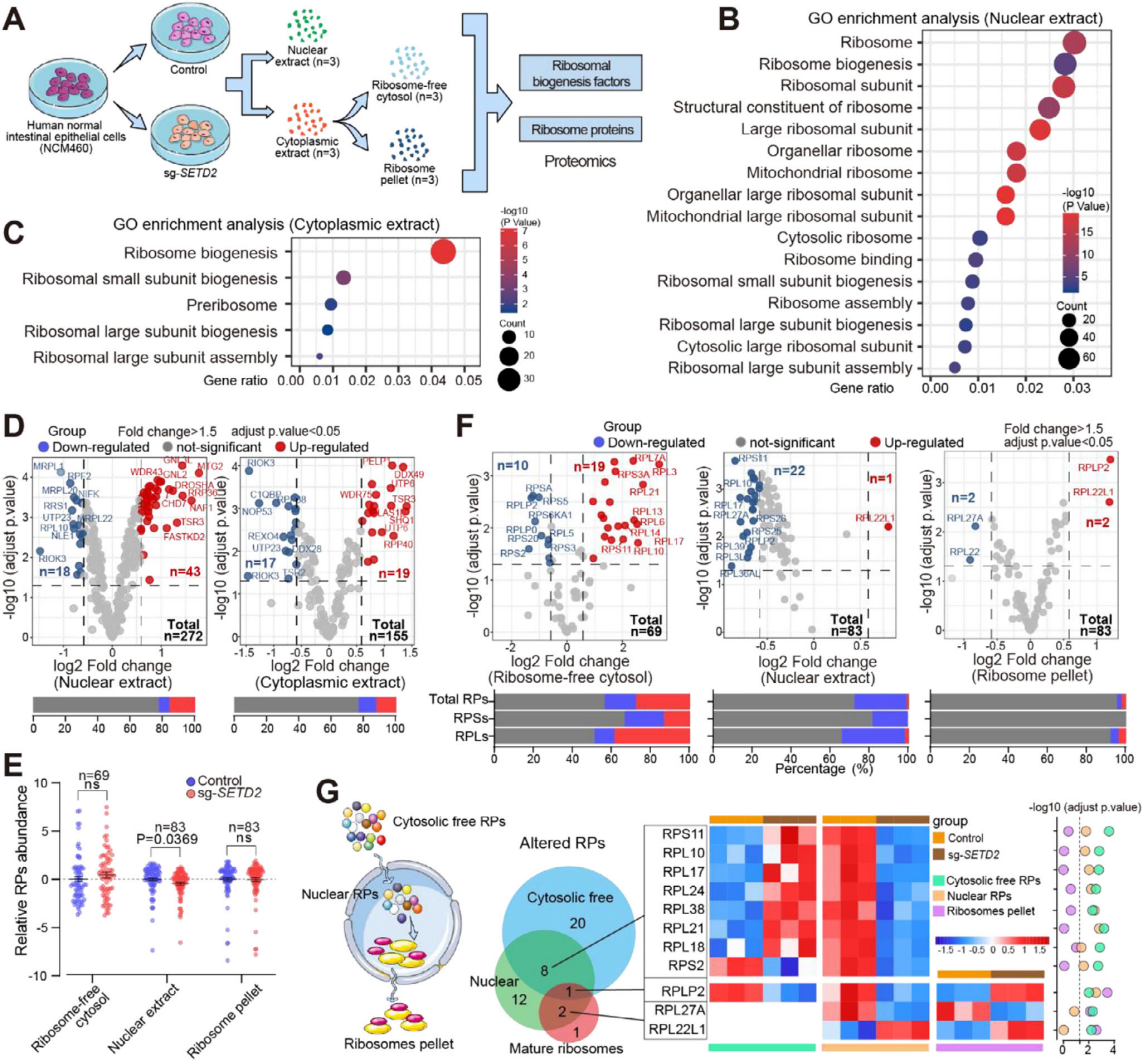
SETD2 ablation leads to recruitment disorders of ribosome biogenesis factors and ribosomal proteins. **(A)** Schematic representation of proteomic analyses. **(B, C)** Significantly altered GO pathways in nuclear and cytoplasmic extract between wildtype and *SETD2* knockout NCM460 cells based on differential proteins. **(D)** Volcano plot of RBFs alterations in different cellular compartments in *SETD2*‐KO IECs compared with wildtype IECs. **(E)** Dot plot shows RP abundance in nuclear extract (n = 83), ribosome‐free cytosol (n = 69), and ribosome pellet (n = 83) between wildtype and *SETD2*‐KO NCM460 cells. **(F)** Volcano plot of RPs alterations in different cellular compartments in *SETD2*‐KO IECs compared with wildtype IECs. **(G)** Venn diagram and heat map showing the RPs displaying altered protein levels in different cellular compartments between wildtype and *SETD2*‐KO IECs. Statistical analyses were performed with a two‐tailed Student's t‐test. Data are represented as mean ± SEM.

Since RBFs' cellular localization is crucial for ribosome biogenesis and functional ribosome composition, we next characterized the alterations in the levels of RBFs and ribosomal proteins (RPs) in normal and SETD2 deficient IECs. To achieve this, we extracted nuclear and cytoplasmic lysates from normal human IECs (NCM460) with or without *SETD2* knockout. Ultracentrifugation was then used to separate the particulate pellet containing ribosomes from the soluble ribosome‐free cytosol [28]. Subsequently, LC‐MS/MS proteomics analyses were conducted to investigate the protein abundance in nuclear, cytoplasmic, ribosome‐free cytosol, and ribosome pellet fractions (Figure 4A; Figure S5A). Proteomics analysis identified 2511 and 1850 differentially expressed proteins (adjusted P. value < 0.05, fold change > 1.5) in nuclear and cytoplasmic lysates, respectively (Figure S5B). GO analyses showed that proteins related to ribosome biogenesis were significantly enriched in both the nuclear extract (Figure 4B) and the cytoplasmic extract (Figure 4C), indicating significant changes of RBFs in both the nucleus and cytoplasm after SETD2 ablation. Specifically, 15.81% of RBFs were upregulated, and 6.62% were downregulated in nuclear extract, and 12.26% of RBFs were upregulated, and 10.97% were downregulated in cytoplasmic extract (Figure 4D).

Then, RP abundances were investigated in nuclear, ribosome‐free cytosol and ribosome pellet fractions (Figure S5C). RP abundance was significantly reduced in the nuclear extract, while the other fractions showed similar RP abundance (Figure 4E). Specifically, 27.71% and 42.03% of RPs were affected in the nuclear extract and ribosome‐free cytosol, respectively, which are pools of free ribosomal proteins. Four RPs were affected in the ribosome pellet, where mature ribosomes are located (Figure 4F). Notably, large ribosomal subunit proteins (RPLs) exhibited more pronounced changes in these fractions compared with small ribosomal subunit proteins (RPSs). During ribosome biogenesis, cytosolic free RPs translocate to the nucleus to assemble into pre‐ribosomes, which are then released back into the cytoplasm. As depicted in Figure 4G, eight RPs (RPS2, RPS11, RPL10, RPL17, RPL24, RPL38, RPL21, and RPL18) were affected in both the ribosome‐free cytosol and nuclear extract, indicating altered distribution of RPs from cytoplasm to nucleus after SETD2 ablation. Moreover, two RPs (RPL27A and RPL22L1) were affected in both the nuclear extract and mature ribosomes, and RPLP2 was affected in all three fractions, indicating altered ribosome assembly after SETD2 ablation (Figure 4G; Figure S5D,E). These results revealed altered RBFs and RPs abundance across different cell fractions, along with changes in ribosome composition in the absence of SETD2.

### 2.5 SETD2 Interacts with RBFs and Governs Ribosome Homeostasis in IECs

**FIGURE 5:**
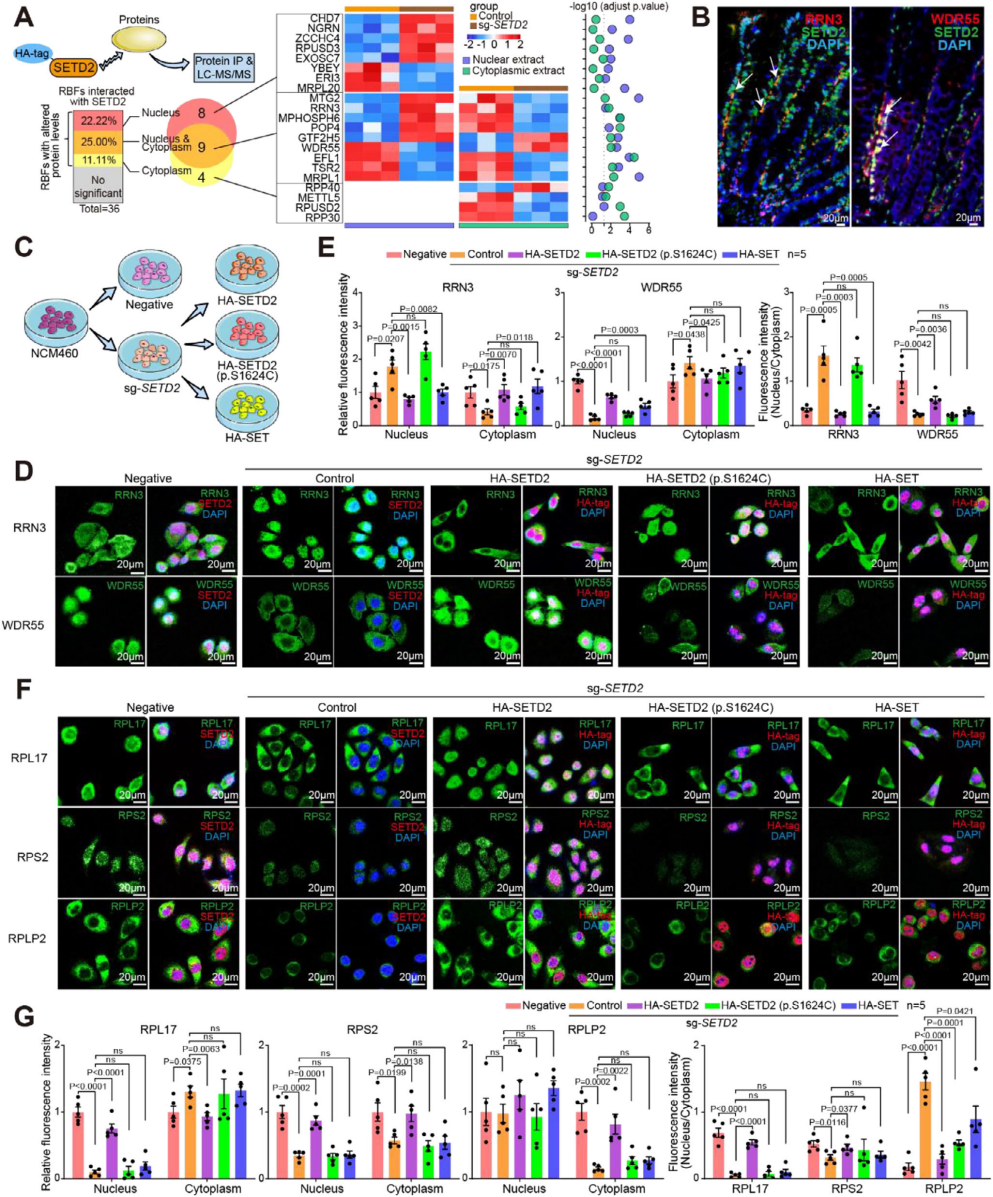
SETD2 interacts with RBFs and governs ribosome homeostasis in IECs. **(A)** Heat map and quantitative analysis (n = 3 per group) of RBFs interacting with SETD2 in different cellular compartments. **(B)** Representative images showing protein expression in colon sections from Setd2^WT^ mice. **(C)** Schematic representation of the cell lines. **(D‐G)** Representative immunofluorescence images and metrics (n = 5 per group) showing histological changes of indicated proteins. Scale bars, 20 µm. Statistical analyses were performed with a two‐tailed Student's t‐test. Data are represented as mean ± SEM.

As a methyltransferase, SETD2 serves as a key regulator of protein structure and activity through its interactions with substrates. To explore the broader functional implications of SETD2 engagement, we next investigated whether the interactions with SETD2 affected the distribution of RBFs and RPs across cellular compartments. To achieve this, we performed immunoprecipitation followed by LC‐MS/MS proteomics analysis using cell lysates extracted from 293T cells with and without HA‐*SETD2* overexpression (Figure S6A). As depicted in Figure 5A, proteomic analysis of HA‐SETD2 immunoprecipitants identified 2301 proteins, including 36 RBFs. However, no RP was annotated within this set of immunoprecipitated proteins. Among these RBFs, eight were dysregulated in the nuclear extract, four were dysregulated in the cytoplasmic extract, and nine were dysregulated in both the nuclear and cytoplasmic extracts. Specifically, four no‐mitochondrial RBFs—RRN3, MPHOSPH6, POP4, and WDR55 exhibited divergent regulation in nuclear and cytoplasmic extracts following SETD2 ablation (Figure 5A; Figure S6B). Notably, immunofluorescence imaging clearly showed that in colon sections of wild‐type mice, RRN3 and WDR55 co‐localized with SETD2 (Figure 5B). In contrast, no such co‐localization was observed for MPHOSPH6 and POP4 (Figure S6C). Subsequently, we eliminated SETD2 and re‐expressed exogenous full‐length SETD2, a methyltransferase‐inactive mutant SETD2 (c.4871C>G, p.S1624C), or the truncated SET domain in NCM460 cells (Figure 5C; Figure S6D). In line with the proteomic data, RRN3 levels increased in the nucleus but decreased in the cytoplasm after SETD2 deletion, while WDR55 levels decreased in the nucleus but increased in the cytoplasm. This indicates significant alterations in the nuclear‐cytoplasmic distribution of RBFs due to *SETD2* deletion. Notably, the truncated SET domain of SETD2, but not the methyltransferase‐inactive mutant, restored the distribution of RRN3 in both the nucleus and cytoplasm, and partially restored the distribution of WDR55 in the nucleus, suggesting a potential role of the SET domain of SETD2 in regulating the nuclear‐cytoplasmic distribution of RBFs (Figure 5D,E). Meanwhile, in line with the proteomic data, RPL17 levels decreased in nucleus and increased in the cytoplasm after *SETD2* deletion, while RPS2 levels decreased in both compartments, and RPLP2 levels decreased in cytoplasm. This indicates significant alterations in the nuclear‐cytoplasmic distribution and abundance of RPs. Notably, neither the truncated SET domain nor the methyltransferase‐inactive mutant of SETD2 was able to restore the distribution of RPL17, RPS2, and RPLP2, highlighting the critical role of full‐length SETD2 in maintaining the proper distribution of RPs (Figure 5F,G). These results revealed the interaction between SETD2 and RBFs, which regulates the distribution of these factors and ribosome homeostasis in IECs.

### 2.6 SETD2 Loss Is Accompanied by Disordered RBFs and RPs in Human Colorectal Tumor

**FIGURE 6:**
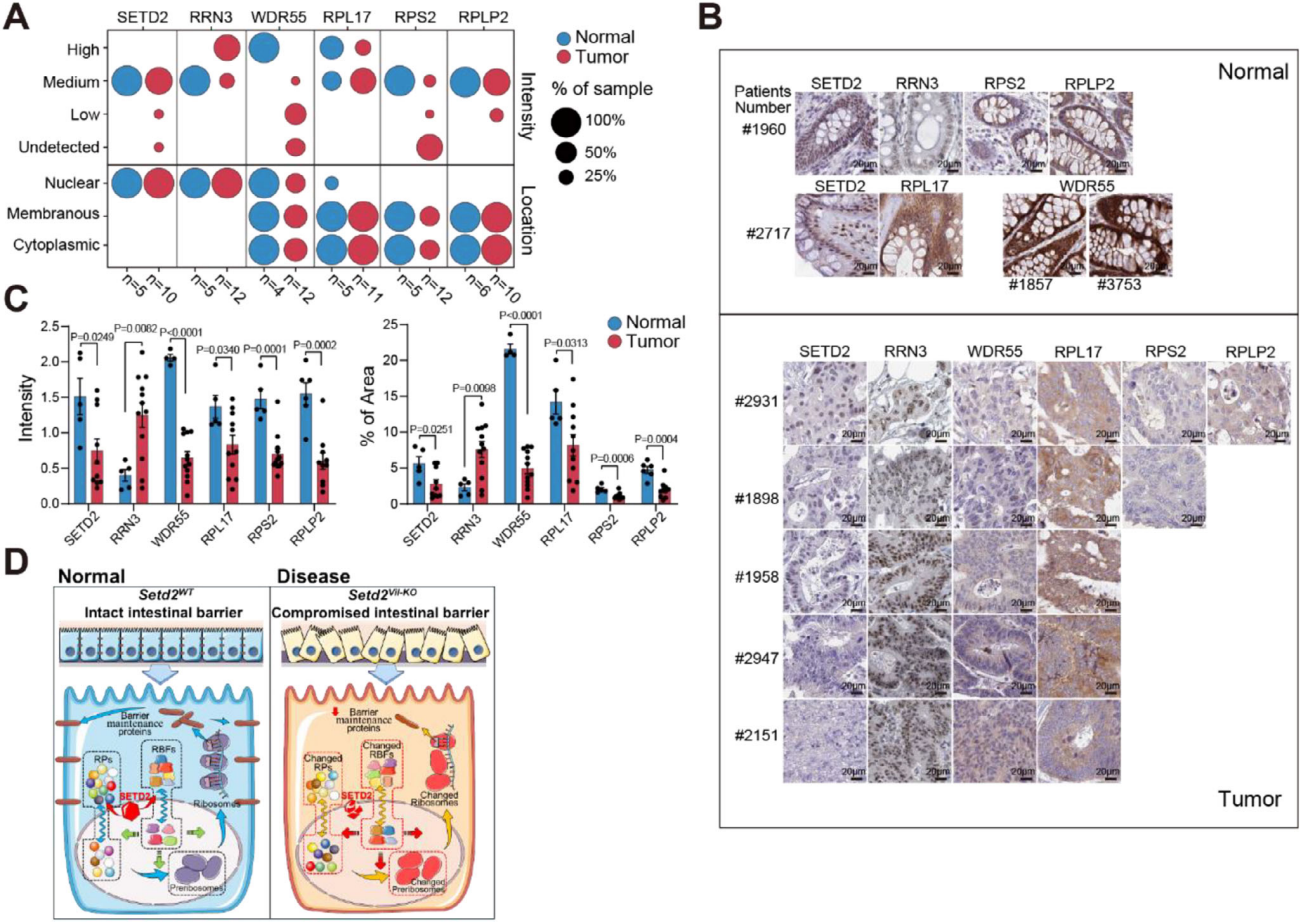
SETD2 loss is accompanied by disordered RBFs and RPs in human colorectal tumor. **(A)** Statistics on staining intensity and subcellular localization in immunohistochemical images derived from the HPA. The sample size for each group is indicated. **(B)** Representative immunohistochemistry images of colorectal tumors from the HPA. Scale bars, 20 µm. **(C)** Quantification of staining intensity and area percentage for the indicated proteins in immunohistochemical images derived from the HPA. Sample size for each group is detailed in Panel A. **(D)** Schematic representing the role of SETD2 in maintaining ribosome homeostasis and intact intestinal barrier. Statistical analyses were performed with a two‐tailed Student's t‐test. Data are represented as mean ± SEM.

To further substantiate the clinical relevance between SETD2 and ribosome biosynthesis in intestinal disease with disordered epithelial barrier, we checked immunohistochemistry images of colorectal tumors from the Human Protein Atlas (HPA, https://www.proteinatlas.org). For RBFs, clinical samples showed concomitant downregulation of SETD2 and WDR55, along with upregulation of RRN3. Notably, subcellular localization statistics revealed reduced nuclear, membrane and cytoplasmic WDR55 staining frequencies. Specifically, 2 of 10 CRC cases exhibited weaker SETD2 staining than normal tissues, while 9 of 12 CRC cases showed high nuclear RRN3 expression, and all CRC cases displayed reduced WDR55 staining, particularly within the nucleus (Figure 6A). Histologically, the nuclear levels of RRN3 increased, whereas those of WDR55 were decreased in CRC patients with low SETD2 expression (Figure 6B,C; Figure S7A). In contrast, no significant changes were observed for MPHOSPH6 and POP4 (Figure S7A–D). For RPs, clinical samples showed concomitant downregulation of SETD2, RPL17, RPS2, and RPLP2. Notably, subcellular localization statistics revealed diminished nuclear RPL17 staining across all 11 CRC cases, as well as decreased membrane and cytoplasmic staining of RPS2 (in 10 of 12 CRC cases) and RPLP2 (in 2 of 10 CRC cases), respectively (Figure 6A). Histologically, we observed a decrease in RPL17 level within the nucleus and reductions in RPS2 and RPLP2 levels in both nuclear and cytoplasmic compartments in SETD2‐low tumor samples (Figure 6B,C; Figure S7A). These findings were consistent with cell immunofluorescence data in Figure 5 and revealed that SETD2 loss is significantly associated with disordered ribosome biogenesis in intestinal disease.

In summary, these findings demonstrate that SETD2 is essential for maintaining the proper distribution of RBFs and RPs, which are crucial for ribosome biogenesis and homeostasis. SETD2 deletion disrupts ribosome biogenesis and leads to an imbalance in ribosome homeostasis, ultimately resulting in reduced translation of barrier maintenance proteins and compromised intestinal barrier in intestinal diseases (Figure 6D).

## 3 Discussion

Strict regulation of cell adhesion and junction in IECs is crucial for functional intestinal barriers and preventing intestinal diseases. Multiple signaling proteins are involved in this process, including small GTP‐binding proteins and tyrosine kinases, such as c‐Src, c‐Yes, and protein kinase C (PKC) [29, 30]. Cytokines such as tumor necrosis factor‐α (TNFα), interferon‐γ (IFN‐γ), and interleukins regulate tight junction by activating the NFκB signaling pathway [31, 32] and regulating myosin light chain kinase (MLCK) transcription [33]. Additionally, post‐translational phosphorylation, particularly of CLDNs, is critical for protein‐protein interactions among cell junction proteins and for maintaining intestinal barrier integrity [34, 35]. In our study, we uncovered a previously unrecognized role of SETD2‐modulated translational regulation in controlling cell adhesion and junction proteins, which advances our understanding of the intestinal barrier and guides the development of novel therapeutic strategies.

The regulation of gene expression is fundamental for life. While transcriptional regulation has long been recognized as crucial, post‐transcriptional regulation, particularly translational regulation, is equally vital. Same as transcription, translation can be regulated globally or at the level of individual transcripts [7]. Such translational alterations can modulate total protein or specific proteins synthesis via preferential mRNA translation [36]. Translation comprises initiation, elongation, termination and ribosome recycling stages, all dependent on functional ribosomes [37]. Defects in ribosome biogenesis and function lead to various diseases known as ribosomopathies [8]. Abnormalities in the ribosome can significantly impact cell fate, causing various ribosome‐related diseases such as COVID‐19 virus infection, bacterial resistance, cardiovascular diseases (CVD), blood diseases, neurodegenerative diseases, and cancer [38, 39, 40], especially intestinal cells and intestinal disease [18]. Mechanisms explaining how aberrant ribosome affects cellular functions include mRNA and cell type sensitivity to ribosome dysfunction [41], selective translation of mRNA determined by ribosome composition [42], and alterations in the p53 pathway [43]. In our study, we observed altered ribosome composition and dysfunctional translation process in compromised intestinal barrier with SETD2 deficiency, suggesting that mRNAs encoding cell adhesion and junction proteins are particularly sensitive to SETD2‐induced disturbances in ribosomal homeostasis. Additionally, further analysis on the post‐translational regulation of proteins associated with intestinal barrier integrity will provide deeper insights into the overall impact of translational regulation on these proteins.

SETD2 was found over two decades ago via its interaction with the Huntingtin (HTT) protein [44, 45], which is closely associated with ribosome‐mediated translational regulation [46, 47]. Increasing evidence indicates that SETD2 is a methyltransferase that can alter the methylation status of H3K36 [48, 49], STAT1 [50], actin [51], α‐tubulin [52], and EZH2 [53], thereby regulating protein structures as well as their functions. Mutations or functional loss of SETD2 have been linked to several diseases, including cancers [54, 55, 56, 57], stem cells differentiation [58], embryonic development [49], lymphocyte development [59], and neurological disorder [60]. Especially, SETD2 has been reported to epigenetically modulate oxidative stress [26] and the RAS/ERK signaling pathway [27] through its histone methyltransferase activity, thereby regulating intestinal epithelial homeostasis and attenuating colonic inflammation and tumorigenesis. Additionally, SETD2 is recognized as a key regulator of Wnt signaling through epigenetic regulation of RNA processing during tissue regeneration and tumorigenesis in the colon [55]. In our study, we identified RBFs that are regulated by SETD2 in IECs. Disordered distributions of both these RBFs and downstream RPs were detected following SETD2 ablation, consistent with observations in clinical histology data. Through these regulations, SETD2 modulates the ribosome biogenesis process to maintain proper translational function, uncovering a previously unrecognized role of SETD2 in regulating translation. Additionally, the truncated SET domain of SETD2 (residues 1460–1720, its main enzymatic active site) can partially substitute for the role of full‐length SETD2 in this process (Figure 5D–G), which points to the potential role of SETD2's methyltransferase activity in regulating RBFs and RPs. Meanwhile, only full‐length SETD2 can elicit the optimal functional effect, indicating the potential role of SETD2's scaffolding and recruiting functions in regulating RBFs and RPs. Thus, further validation of the specific methylation sites of these RBFs, together with characterization of SETD2's scaffolding and recruiting function, will deepen our understanding of SETD2's role in translational regulation.

In eukaryotes, ribosome biogenesis is a complex and multistep process that involves over 280 RBFs, consecutively in the nucleoli, the nucleoplasm, and the cytoplasm [61]. It is worth pointing out that methyltransferase SET domain of SETD2 partially contributes to its regulatory role on RBFs, and only full‐length SETD2 ensures proper distribution of RPs. These results suggest that SETD2 might regulate RBFs through both methyltransferase‐dependent and ‐independent mechanisms. Further investigation is needed to determine the specific interaction sites between SETD2 and these RBFs, as well as the molecular functions of SETD2 in this context. Notably, as shown in Figure 1H, seven RBFs exhibited consistent changes in both their mRNA and protein levels, suggesting a potential role of SETD2 in regulating the transcription of these genes. The possible transcriptional regulatory functions of SETD2 on RBFs require further study.

Collectively, we demonstrated that the dysregulation and recruitment disorder of ribosome biogenesis factors induced by SETD2 ablation affects the composition and distribution of ribosomal proteins, thereby disrupting the ribosome homeostasis in IECs. Consequently, disrupted ribosome homeostasis leads to translational disorder and reduced translational efficiency of mRNAs encoding cell adhesion and junction proteins, thereby compromising the stability of the epithelial barrier. Clinical specimens further confirm the abnormal intensity and distribution of RBFs and RPs in patients with low expressed SETD2. Our findings uncover a regulatory mechanism by which SETD2 modulates ribosome homeostasis and reveals a previously unappreciated role of ribosome biogenesis in safeguarding epithelial barriers. The *Setd2* mutant mice would serve as a genetically engineered mouse model for pre‐clinical research on intestinal diseases with ribosomal disorder.

## 4 Experimental Methods

### 4.1 Mice

*Setd2^fl/fl^* mice were generated as previously reported [62]. *Villin^Cre^* mice were purchased from Shanghai Biomodel Organism Co. Mice with accurately confirmed genotypes were used in experiments. Randomization was not applied to the multi‐omics study, whereas data assessments, including histological scoring, were conducted in a blinded manner. Our study is not sex‐ or gender‐based. Order of treatments and measurements, and cage location were used to minimize potential confounders. All researchers were aware of the group allocation at all stages of the experiment. All mice were maintained in a specific‐pathogen‐free (SPF) facility and were housed at a temperature of 25°C in a humidity‐controlled environment with free access to food and water in a 12 h light/dark cycle. To minimize distress, this study employed carbon dioxide asphyxiation for euthanasia of mice. All animal experimental procedures were reviewed and approved by the Institutional Animal Care and Use Committee (IACUC) of Shanghai Jiao Tong University (NO. 2023008). Diets for all animal experiments were purchased from SLACOM (Catalogue number: P1101F‐25).

### 4.2 Induction of Epithelial Damage

To induce epithelial damage, 8‐week‐old Setd2^WT^ and *Setd2^Vil‐KO^* mice were administered 2% dextran sodium sulfate (DSS; MP Biomedicals) in drinking water for 5 days, followed by a 5‐days recovery period with regular drinking water.

### 4.3 Mouse Intestinal Permeability Assay

Mice were administered 40 mg 4‐kDa FITC‐labeled dextran (46944; Sigma–Aldrich) per 100 g body weight after a 4‐h water and food deprivation. Four hours later, blood was obtained through retro‐orbital bleeding and subsequently centrifuged at 2000 rpm for 10 min. Serum samples were assayed for FITC fluorescence at 485 nm excitation and 530 nm emission using a TECAN Infinite M200 plate reader. Concentrations of FITC‐dextran were quantified based on a standard curve.

### 4.4 Isolation of Intestinal Epithelial Cells

Mice were sacrificed, and colonic tissues were dissected out, opened longitudinally, and extensively washed with cold PBS to remove feces. Colonic tissues were sectioned into 1 mm^3^ fragments and incubated in PBS containing 30 mm EDTA at 37°C for 10 min. After 10 min, the EDTA solution was replaced with ice‐cold PBS, followed by shaking for 30 s. Supernatants were collected and centrifuged at 1200 rpm for 5 min at 4°C. The pellet contained isolated intestinal epithelial cells. The purity of colonic epithelial cells isolated via EDTA‐based digestion was 90%–95%, as verified by EpCAM staining (Invitrogen #25‐ 5791‐80) [26].

### 4.5 Immunofluorescent and Immunohistochemistry (IHC) Assays

Tissues were fixed in 10% buffered formalin, and fixed tissues were sectioned for hematoxylin and eosin (H&E) staining. For IHC staining, paraffin‐embedded tissues were deparaffinized, rehydrated, and subjected to a heat‐induced epitope retrieval step by treatment with 0.01 m sodium citrate (pH 6.0). Endogenous peroxidase activity was blocked with 0.3% (v/v) hydrogen peroxide in distilled water. Cells were plated on cover slides in 24‐well plates and allowed to grow for 24 h. The slides were then fixed in 4% formalin for 15 min at 4°C and rinsed with PBS three times. The tissue sections and cell cover slides were first treated with 0.5% Triton X‐100 for 10 min and blocked with 10% goat serum at room temperature for 1 h. Primary antibody incubations were performed overnight at 4°C. After extensive washing with PBS, secondary antibody was applied to the sections at room temperature for 1 h. Primary antibody against SETD2 (Abclonal, A23237), ZO‐1 (Invitrogen, 40–2200), CLDN2 (abcam, ab53032), E‐cadherin (CST, #3195T), MPHOSPH6 (Santa Cruz, sc‐393429), POP4 (Biorbyt, orb312651), RRN3 (Santa Cruz, sc‐390464), WDR55 (Santa Cruz, sc‐514225), RPL17 (Santa Cruz, sc‐515904), RPLP2 (Novus, NBP2‐15104), RPS2 (Santa Cruz, sc‐130399) and HA‐tag (CST, #3724T) were used.

### 4.6 Western Blot Analysis and Antibodies

Cells were lysed with 100–300 mL RIPA lysis buffer (50 mm Tris‐Base, 150 mm NaCl, 5 mm EDTA, 1% NP‐40, 0.1% SDS, pH 7.4) supplemented with protease and phosphatase inhibitors (Millipore). From each sample, 20–50 mg of total protein was separated by 8%–12% SDS‐PAGE gels and transferred onto nitrocellulose membranes (GM). Membranes were blocked in 5% BSA in TBS for 1 h at room temperature and then incubated with primary antibodies overnight at 4°C, washed in TBS containing 1% Tween20, incubated with a horseradish peroxidase (HRP)‐conjugated secondary antibody for 1 h at room temperature, and developed by ECL reagent (Thermo Fisher Scientific). The immunoblots were quantified by Bio‐Rad Quantity One version 4.1 software. Primary antibodies against SETD2 (Abclonal, A23237), GAPDH (CST, 2118T), Histon 3(CST, 4499T), HA‐tag (CST, #3724T), PKCθ (Santa Cruz, sc‐1680), NDUFB9 (abcam, ab200198) were used.

### 4.7 Immunoprecipitation

Cells were washed with ice‐cold PBS and lysed in RIPA lysis buffer supplemented with protease and phosphatase inhibitors (Millipore) at 24 h after transfection. Cell lysates were incubated with primary antibodies overnight at 4°C. Protein A/G Magnetic Beads (MCE, #HY‐K0202) were then added, and the lysates were incubated for another 4 h at 4°C. The immunoprecipitants were washed four times with the lysis buffer and stored at −80°C. Antibodies used in the co‐immunoprecipitation experiments were as follows: SETD2 (Abclonal, A23237), HA‐tag (CST, #3724T), and DYKDDDDK (Flag)‐tag (CST, #14793T).

### 4.8 RNA‐Seq Library Construction and Sequencing

IECs from mice were isolated for RNA preparation. The complementary DNA (cDNA) libraries were prepared using the NEBNext TM Ultra Directional RNA Library Prep Kit, NEBNext Poly (A) mRNA Magnetic Isolation Module, and NEBNext Multiplex Oligos according to the manufacturer's instructions. The products were purified and enriched by PCR to create the final cDNA libraries and quantified by Agilent2200. The barcoded cDNA libraries were pooled in equimolar ratios and subjected to 150‐bp paired‐end sequencing on a single lane of an Illumina HiSeq X Ten platform.

### 4.9 Ribo‐Seq Library Construction and Sequencing

A total of 10^7^ cells were washed twice with ice‐cold PBS containing 100 µg/mL cycloheximide (Adooq, 66‐81‐9). Ribosome profiling was performed using Epi Ribosome Profiling Kit (Epibiotek, R1814). Subsequently, RPFs (ribosome‐protected RNA fragments) were extracted using RNA clean&ConcentratorTM‐5 kit (ZYMO, R1016). EpiTM RiboRNA Depletion Kit (Human/Mouse/Rat) (Epibiotek, R1805) was used for rRNA depletion. Sequencing libraries were constructed using QIAseq miRNA Library kit (QIAGEN, 1103679).

### 4.10 Routine Data Process of RNA‐Seq and Ribo‐Seq Data

The raw RNA‐seq and Ribo‐seq data quality were evaluated first using FastQC (v0.11.9), and then reads with low quality and more than 10% Ns were filtered using Fastp (v0.20.1). Adapter sequences were trimmed from raw ribosome profiling data using cutadapt software (https://code.google.com/p/cutadapt/) [63]. Meanwhile, reads with lengths between 25 and 35 bp were kept for downstream analysis. To exclude rRNA and tRNA reads, we aligned sequences to the SILVA rRNA database (https://www.arb‐silva.de) and Genomic tRNA database (http://gtrnadb.ucsc.edu, mm10‐tRNAs.fa) using bowtie2 (v2.2.6) with default parameters. The remaining reads were aligned to GRCm38/mm10 using HISAT2 (v2.2.1) with GENCODE M19 transcript coordinates. Reads mapping to UTR regions and ncRNAs, as well as genes with zero counts were filtered out prior to analysis. The trinucleotide periodicity of ribosomes and codon usage frequency were estimated using revised riboWaltz package [64]. Read counts were calculated using featureCounts software. Raw counts were further normalized as TPM values using TPM function in DESeq2 package. Translational efficiencies were determined as the ratio of (normalized abundance determined by ribosome profiling)/ (normalized abundance determined by RNA‐seq) as previously reported [65].

### 4.11 Proteomics Analysis

One hundred micrograms of extracted proteins were reduced with 10 mm dithiothreitol and alkylated with 25 mm iodoacetamide. The samples were then incubated with trypsin (enzyme to protein ratio 1:50) overnight for digestion. For IP‐MS experiment, about 1 mg of cell lysates were subjected to Protein A/G Magnetic Beads (MCE, # HY‐K0202). The beads were washed three times with PBS before digestion. 100 µL of elution buffer (2 m Urea, 10 mm DTT, 50 mm Tris‐HCl pH 8) were added to beads, after 20 min incubation at RT, the supernatant was separated and alkylated with 25 mm iodoacetamide. Protein on the beads were partially digested for 1 h and combined to the supernatant above. After overnight digestion, the resulting peptides were desalted and dried for LC‐MS/MS measurement. The peptides were analyzed using a NanoElute LC system coupled to a timsTOF Pro mass spectrometer (Bruker Daltonics). Two hundred nanograms of peptides were loaded onto a 15 cm Aurora Elite column, and eluted by a 90 min gradient starting at 2% acetonitrile and stepwise increase to 22% acetonitrile in 75 min, 37% acetonitrile in 5 min, and 80% acetonitrile in 5 min. The mass spectrometer was operated in DIA‐PASEF mode with a 22 Th isolation window covering an m/z range from 344 to 1158 Th. The capillary voltage was set to 1500 V, and the collision energy was linearly increased from 20 to 59 eV. DIA‐NN (version 1.8.1) was used to analyze the MS data. The default settings were kept except that we changed the maximum number of missed cleavages to 2. MS Spectra were searched against a human proteome FASTA file (20577 entries) and a mouse proteome FASTA file (21986 entries).

### 4.12 Pathway Enrichment Analysis

The GO/KEGG enrichment analysis was executed using clusterProfiler package (v.4.5.0) through Hiplot Pro (https://hiplot.com.cn/), a comprehensive web service for biomedical data analysis and visualization. Gene expression data of mRNA and protein levels were used to obtain NES over the active GO/KEGG biological processes using GSEA software (v.4.1) (http://software.broadinstitute.org/gsea/index.jsp). GO and KEGG enrichment terms were visualized as plots of NES for mRNA and protein datasets. GO gene sets belonging to Mus musculus used in this study were integrated from AmiGO database (http://amigo.geneontology.org/amigo/landing).

### 4.13 Plasmids, Transfection, and Lentivirus

cDNA of human SETE2 and truncated SET domain of SETD2 (residues 1495–1693) were generated by polymerase chain reaction. h‐SETD2 methyltransferase mutant (c.4871C > G, p.S1624C [66]) plasmid was a kind gift from Guan Ning Lin (Shanghai Jiao Tong University, Shanghai, China). cDNA fragments encoding wild‐type SETD2 and the SETD2 p.S1624C mutant were cloned into pCMV6‐Entry vector with HA‐tags. SgRNA sequences for SETD2 and scramble sgRNA were cloned into lentiviral vector lentiCRISPRv2 (sg‐SETD2‐C1: AGAAATCAGTCGGCAGGACA; sg‐SETD2‐D1: AAGTATCTAGCTTCTAACCA). For transfection, cells were transfected with the jetPRIME transfection reagent (Polyplus) according to the manufacturer's instructions. All the constructions generated were confirmed by DNA sequencing. Lentiviral packaging plasmids pCMV‐DR8.8 and pMD2.G were co‐transfected with the backbone plasmid into 293T cells for virus production. Stable transfected cell lines were established by subjecting cells to selection in culture medium containing 2 µg/mL puromycin.

### 4.14 Cell Lines and Culture

NCM460 and 293T cell lines were obtained from the American Type Culture Collection (ATCC) and authenticated via short tandem repeat (STR) profiling. NCM460 and 293T cells were cultured in RPMI‐1640 and DMEM, respectively, supplemented with 10% FBS (Thermo), 100 U/mL penicillin, and 0.1 mg/mL streptomycin (Thermo) at 37°C in a humidified 5% CO2 atmosphere.

### 4.15 OPP Protein Synthesis Assay

Protein synthesis was detected using the Click‐iT Plus OPP Alexa Fluor 488 Protein Synthesis Assay Kit (Invitrogen #C10456) according to the manufacturer's recommendations. After being cultured under the indicated conditions, cells were incubated in media containing 20 µm OPP for 30 min at 37°C. Cells were then fixed with 3.7% formaldehyde, permeabilized with 0.5% Triton X‐100, and incubated with the OPP reaction cocktail for 30 min at room temperature, protected from light. After nuclear staining, the cells were processed for subsequent imaging and analysis.

### 4.16 Polysome Profiling Analysis and Quality Control

Cells were treated with 100 µg/mL cycloheximide (Sigma) for 15 min at 37°C. Then, cells were collected and lysed on ice with lysis buffer. Cell debris was removed by centrifugation at 13 000 g for 10 min at 4°C. A 10%–50% (w/v) sucrose density gradients were freshly prepared in SW41 ultracentrifuge tubes (Backman) using a Gradient Master (BioComp Instruments). Then, 1 mL of supernatant was loaded onto sucrose gradients followed by centrifugation for 3 h at 38 000 g 4°C. Gradients were fractionated and monitored at absorbance 254 nm (BioRad). Non‐ribosome, 40S–80S and polysome fractions were pooled, and RNA was isolated using TRIzol reagent (Thermo Fisher) following the manufacturer's instructions.

### 4.17 Primers

RT‐qPCRs were performed using oligonucleotides corresponding to human rDNA [67] and the CDS regions of ANXA1, ANXA5, and CLDN2. The forward primers were: ETS1, 5’‐GAGGTTGGGCCTCCGGATGC‐3’; ETS3, 5’‐CCTCTGACGCGGCAGACAGC‐3’; 5.8S1, 5’‐CACTTCGAACGCACTTGCGG‐3’; 18S1, 5’‐GTTCAAAGCAGGCCCGAGCC‐3’; F‐ANXA1, 5’‐ CGAAACAATGCACAGCGTCA‐3’; F‐ANXA5, 5’‐ TTCAGGGTACTACCAGCGGA‐3’; F‐ CLDN2, 5’‐ AAAGACAGAGTGGCGGTAGC‐3’. The reverse primers were: ETS2, 5’‐ACGCGCGAGAGAACAGCAGG‐3′; ETS4: 5’‐CTCCAGGAGCACCGCAAGGG‐3’; 5.8S2, 5’‐CTGCGAGGGAACCCCCAGCC‐3’; 18S2, 5’‐AGCGGCGCAATACGAATGCC‐3’; R‐ANXA1, 5’‐ AGCCTGAAATAAAGCCTGAGCA‐3’; R‐ANXA5, 5’‐ TCAGTTCCAAGGCCCTTCAT‐3’; R‐CLDN2, 5’‐ AGCTATCAGGGAGAACAGGGA‐3’.

### 4.18 RT‐qPCR

Total RNA was isolated from cultured cells or polysome profiling fractions with Trizol reagent (Invitrogen). cDNA was synthesized by reverse transcription using the Prime Script RT reagent kit (TaKaRa). Real‐ time PCR was performed using SYBR Green Realtime PCR Master Mix (Thermo). For rRNA analysis, normalization was performed using primers 18S1 and 18S2, which primarily amplify the cDNAs corresponding to mature 18S rRNA.

### 4.19 Quantification and Statistical Analysis

Differences between the two groups were assessed using two‐tailed unpaired Student's t‐tests. The Pearson correlation coefficient was used to analyze the strength of the association. A significant difference was defined as P. value < 0.05. GraphPad Prism v8.4.2.679 was used for all statistical analyses.

## Author Contributions

Conceptualization: L.L. and X.D. Data curation: H.R., A.W., Y.X., W.F., C.M., Z.W., W.Z., and W.S. Formal analysis: H.R. and A.W. Software: A.W. Visualization: H.R. Supervision: L.L. and X.D. Writing – original draft: H.R. Writing – review & editing: L.L. and X.D. W.Q.G. and X.X. assisted in the valuable discussion.

## Ethical Statement

All animal experimental procedures were reviewed and approved by the Institutional Animal Care and Use Committee (IACUC) of Shanghai Jiao Tong University (NO. 2023008).

## Conflicts of Interest

The authors declare no conflicts of interest.

## Supporting information




**Supporting File 1**: advs73693‐sup‐0001‐SuppMat.pdf.

**Supporting File 2**: advs73693‐sup‐0002‐Table S1.xlsx.

## Data Availability

RNA‐seq and rib‐seq data have been deposited in the GEO under accession number GSE 151968 and GSE 276958 respectively. The mass spectrometry proteomics data have been deposited to the ProteomeXchange Consortium via the PRIDE partner repository and iProX database with dataset identifier PXD055747 and PXD070208 [68]. The gene expression data was downloaded from gene Expression Omnibus (GEO) and The Cancer Genome Atlas (TCGA). We checked immunohistochemistry images of normal tissue (https://v24.proteinatlas.org/humanproteome/tissue) and colorectal tumors (https://v24.proteinatlas.org/humanproteome/cancer) from the Human Protein Atlas (HPA) [69], in which staining is reported in four levels, (high, medium, low, and not detected), and cell distribution patterns (nuclear, membrane, and cytoplasmic localization). We have obtained formal copyright permission to use their colorectal tumor images.
