# Supplementary material for: Ribosome Homeostasis Regulated by SETD2 Preserves Intestinal Epithelial Barrier
Source: Adv Sci (Weinh). 2026 Jan 4;13(16):e08168. doi: 10.1002/advs.202508168 (PMC13042805; doi:10.1002/advs.202508168)
Supplement: Supplementary file 1 — Supporting File 1: advs73693‐sup‐0001‐SuppMat.pdf. [file ADVS-13-e08168-s001.pdf]

Supporting Information

Title

Ribosome Homeostasis Regulated by SETD2 Preserves Intestinal Epithelial Barrier

Authors

Hanyu Rao[1,2,Δ], Aiting Wang[2,3,Δ], Yue Xu[1], Wenxin Feng[1], Chunxiao Ma[1], Ziyi Wang[1], Wei Zhang[1], Wenqiong Su[2,3], Xiuying Xiao[4], Wei-Qiang Gao[1], Xianting Ding[2,3], Li Li[1,2,]**

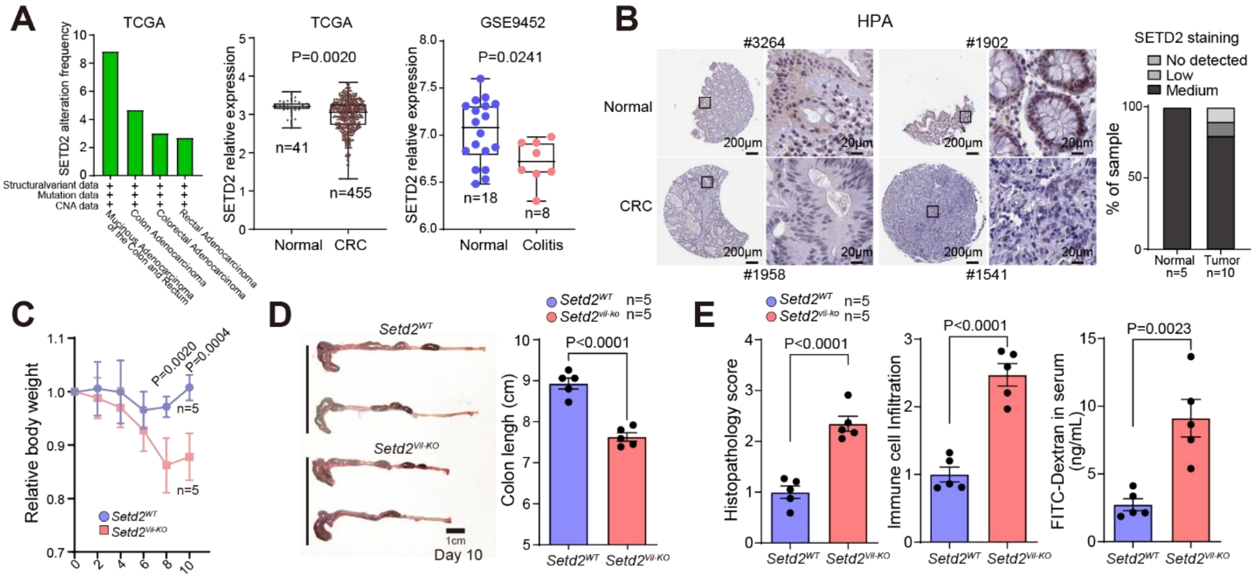

Figure S1. SETD2 deficiency exacerbates the impairment of intestinal barrier.

(A) Boxplot of SETD2 alteration frequency in colorectal tumors and its expression levels in CRC, IBD specimens and healthy controls. **(B)** Immunohistochemistry images of colorectal tumors from the Human Protein Atlas. Losses of body weight **(C)** and colon length **(D)** are recorded (n=5 per genotype). Scale bars: 1 cm. **(E)** Quantification of epithelial damage, immune cell infiltration and intestinal permeability are shown on the right (n=5 per genotype). Statistical comparisons were made using a two-tailed Student's t test. Data are represented as mean ± SEM.

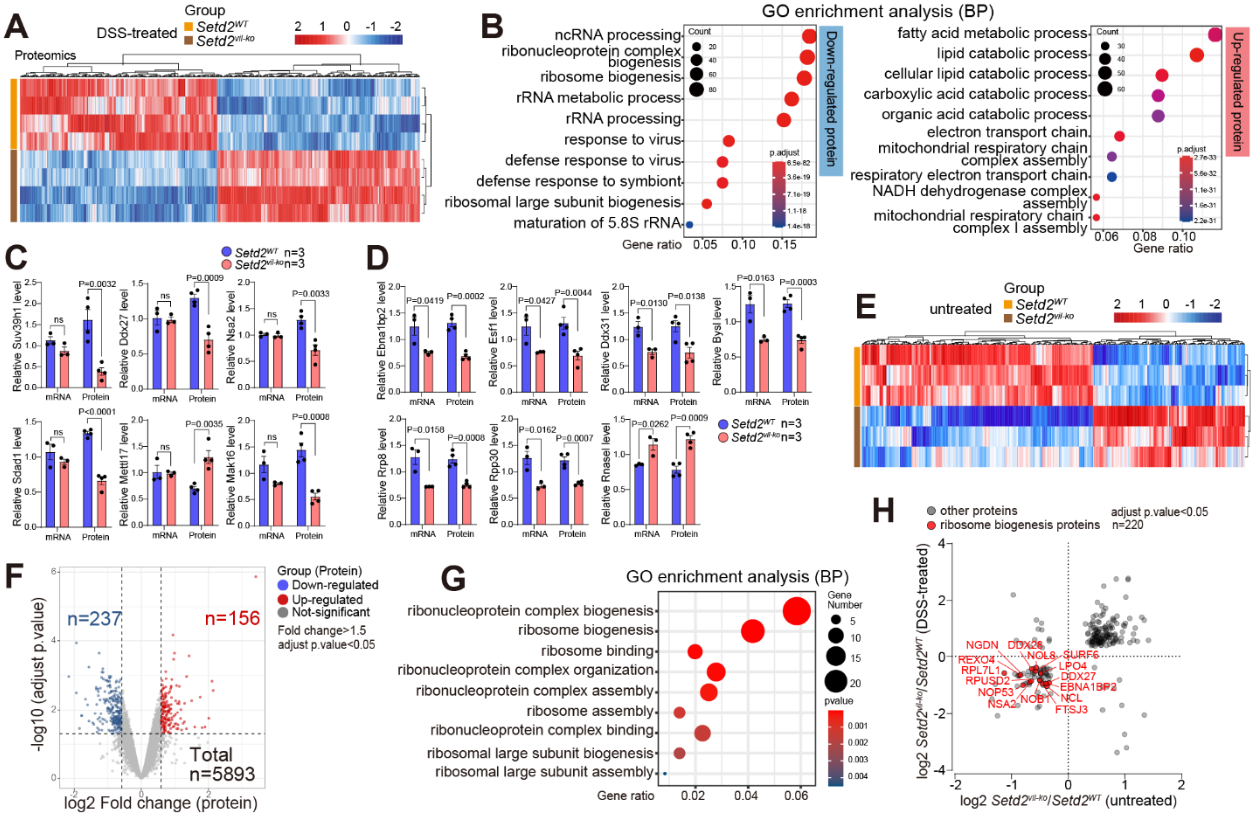

Figure S2. SETD2 deficiency results in dysregulated ribosome biogenesis factors in IECs. **(A)** Heat map of the altered proteins (n=4 per genotype) between IECs from DSS-treated *Setd2*$^{Vil\text{-}KO}$ and *Setd2*^{WT} mice. **(B)** Significantly altered GO pathways in IECs following *Setd2* deletion in DSS-treated mice based on differential proteins. **(C, D)** Relative mRNA (n=3 per genotype) and protein (n=4 per genotype) levels of RBFs between IECs from *Setd2*$^{Vil\text{-}KO}$ and *Setd2*^{WT} mice. **(E)** Heat map of the altered proteins (n=3 per genotype) between IECs from untreated *Setd2*$^{Vil\text{-}KO}$ and *Setd2*^{WT} mice. **(F)** Volcano plot of protein alterations in IECs from untreated *Setd2*$^{Vil\text{-}KO}$ versus *Setd2*^{WT} mice. **(G)** Significantly altered GO pathways in IECs following *Setd2* deletion in untreated mice based on differential proteins. **(H)** Significantly changed proteins (adjusted p-value < 0.05) following *Setd2* deletion under both DSS-treated and untreated conditions. RBFs are highlighted in red. Statistical comparisons were made using a two-tailed Student's t test. Data are represented as mean ± SEM.

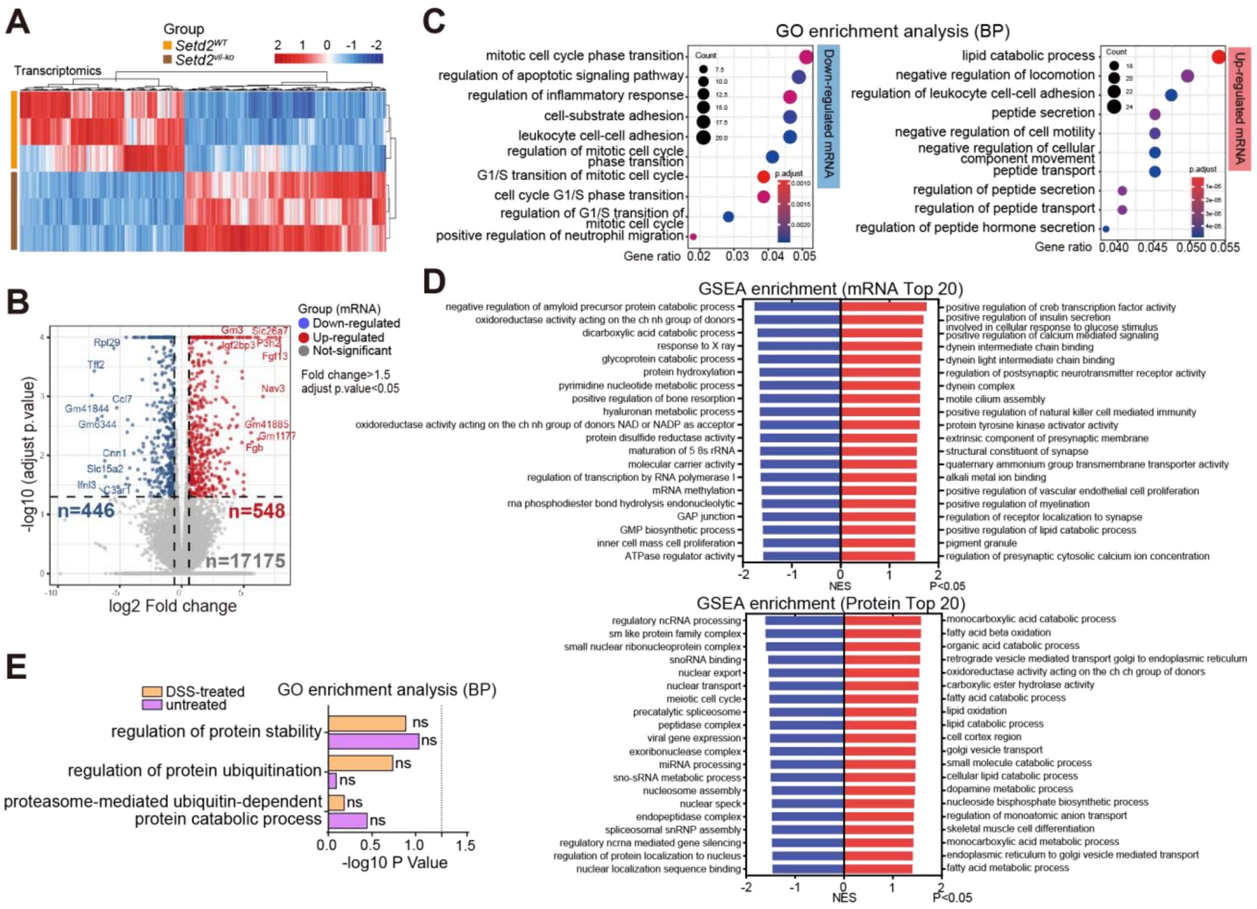

Figure S3. Transcriptomics and proteomics analyses of IECs from *Setd2$^{Vil\text{-}KO}$* and *Setd2^{WT}* mice.

(A) Heat map of the altered mRNAs (n=3 per genotype) between IECs from DSS-treated *Setd2$^{Vil\text{-}KO}$* and *Setd2^{WT}* mice. **(B)** Volcano plot of mRAN alterations in IECs following *Setd2* deletion. **(C)** Significantly altered GO pathways in IECs following *Setd2* deletion based on differential mRNAs. **(D)** GSEA analysis of up-regulated and down-regulated pathways in IECs following *Setd2* deletion based on GO subset in AmiGO database. (**E**) GO pathways analysis of protein alterations related to protein stability and degradation between IECs from DSS- and un- treated *Setd2$^{Vil\text{-}KO}$* versus *Setd2^{WT}* mice. Statistical comparisons were made using a two-tailed Student's t test.

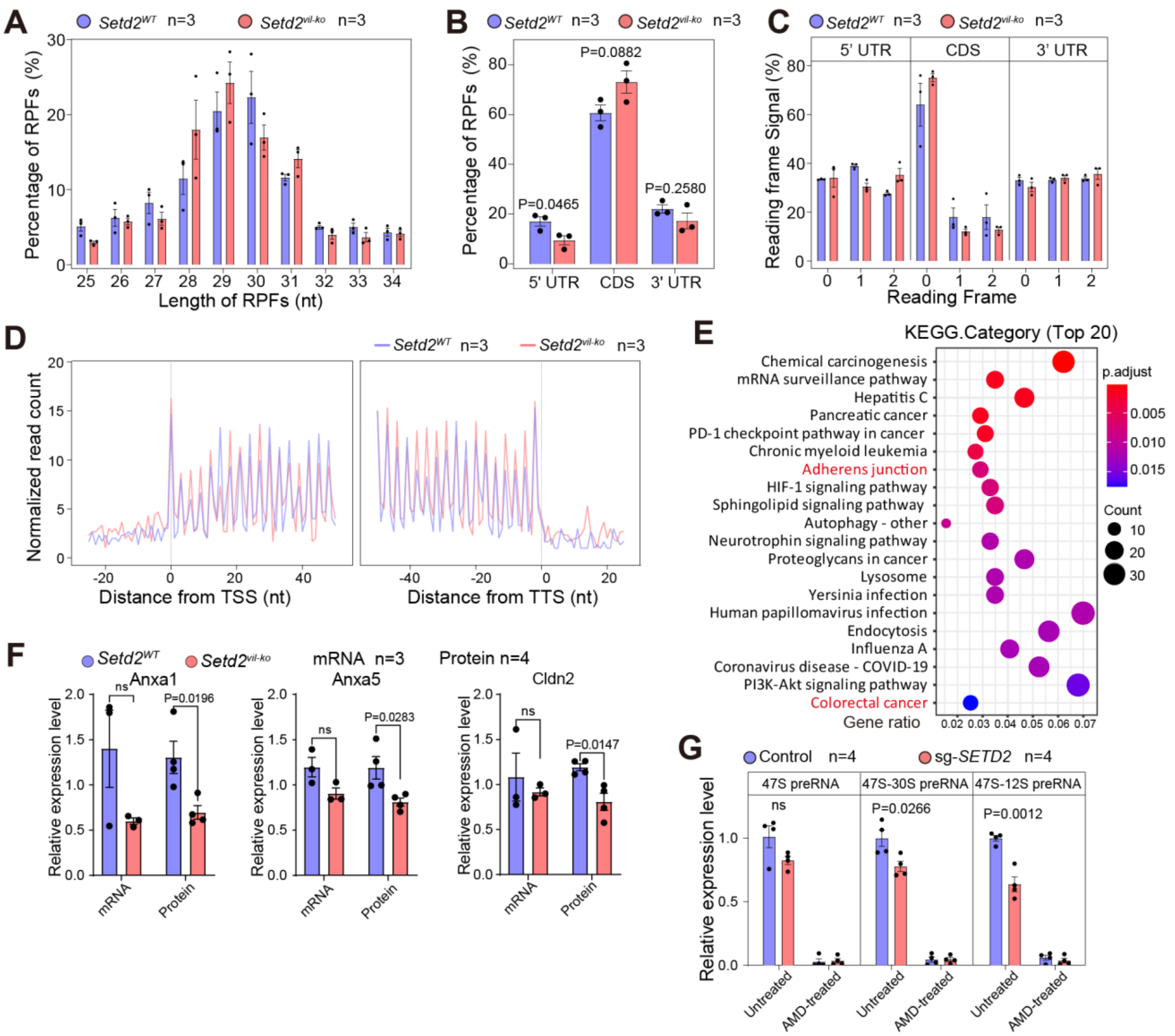

Figure S4. SETD2 deficiency leads to dysregulated translational efficiency in IECs.

(A) Length distribution of Ribo-seq ribosome-protected fragments (RPFs). (**B**) The proportion of RPFs within annotated genes. (**C**) Reading frames of optimally mapped Ribo-seq reads within annotated genes. **(D)** Distribution of optimally mapped reads along the CDS within each codon. Each read was represented by a specific P-site position depending on its fragment length. TSS, translation start site; TTS, translation termination site. **(E)** Significantly altered KEGG pathways in IECs following *Setd2* deletion based on genes with altered TE. **(F)** Relative mRNA (n=3 per genotype) and protein (n=4 per genotype) levels of indicated genes between IECs from *Setd2*$^{Vil\text{-}KO}$ and *Setd2*^{WT} mice. (**G**) RT-qPCR analyses using primers ETS1 and ETS2 to quantify 47S pre-rRNA, primers ETS3 and ETS4 to quantify 47S–30S pre-rRNAs, and primers 5.8S1 and 5.8S2 to

quantify 47S–12S pre-rRNAs, transcription inhibitor actinomycin D (AMD) was used as a positive control. Statistical comparisons were made using a two-tailed Student's t test. Data are represented as mean ± SEM.

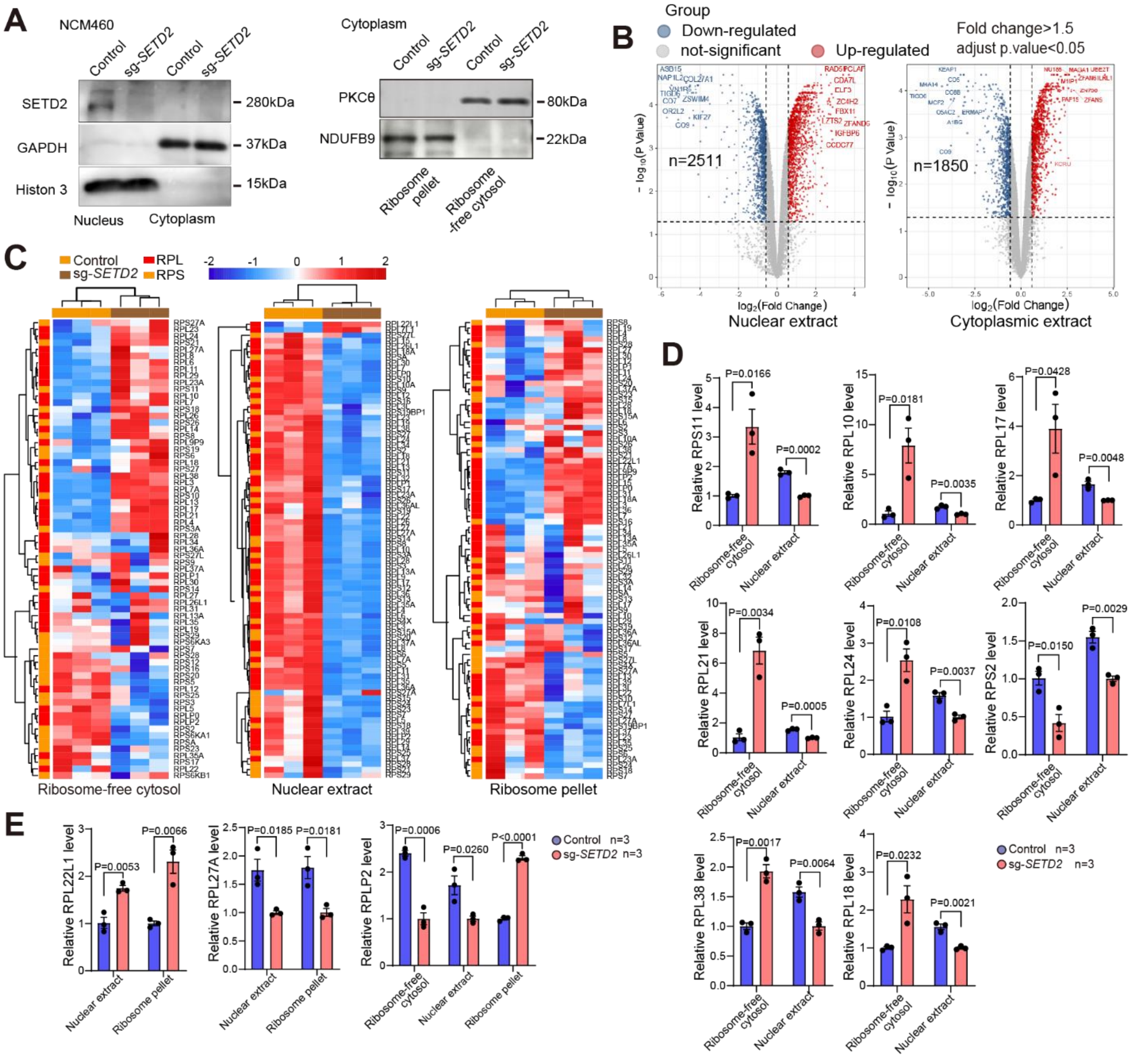

Figure S5. SETD2 regulates the distribution of ribosome biogenesis factors and ribosomal proteins.

(A) The protein levels of indicated genes in different cellular compartments extracted from wildtype and *SETD2*-KO IECs. **(B)** Volcano plot of protein alterations in nuclear and cytoplasmic extract following *SETD2* ablation. **(C)** Relative RP abundance in different cellular compartments extracted from wildtype and *SETD2*-KO IECs. **(D, E)** Relative protein levels of RPs in different

cellular compartments between wildtype (n=3) and *SETD2*-KO (n=3) IECs. Statistical comparisons were made using a two-tailed Student's t test. Data are represented as mean ± SEM.

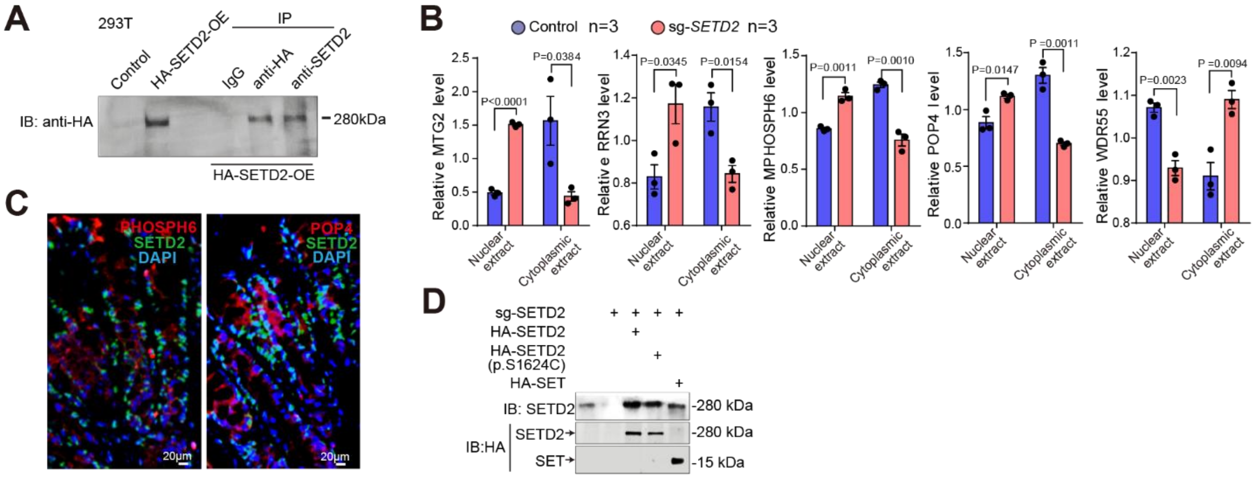

Figure S6. SETD2 interacts with RBFs and governs ribosome homeostasis in IECs.

(A) Co-immunoprecipitation experiment on HA-SETD2-overexpressed 293T cells. **(B)** Quantitative analysis (n=3 per group) of RBFs interacting with SETD2 in different cellular compartments. **(C)** Representative images showing protein expression in colon sections from *Setd2*^{WT} mice. Scale bars, 20 μm. **(D)** IB analysis of indicated proteins in NCM460 cells. Statistical comparisons were made using a two-tailed Student's t test. Data are represented as mean ± SEM.

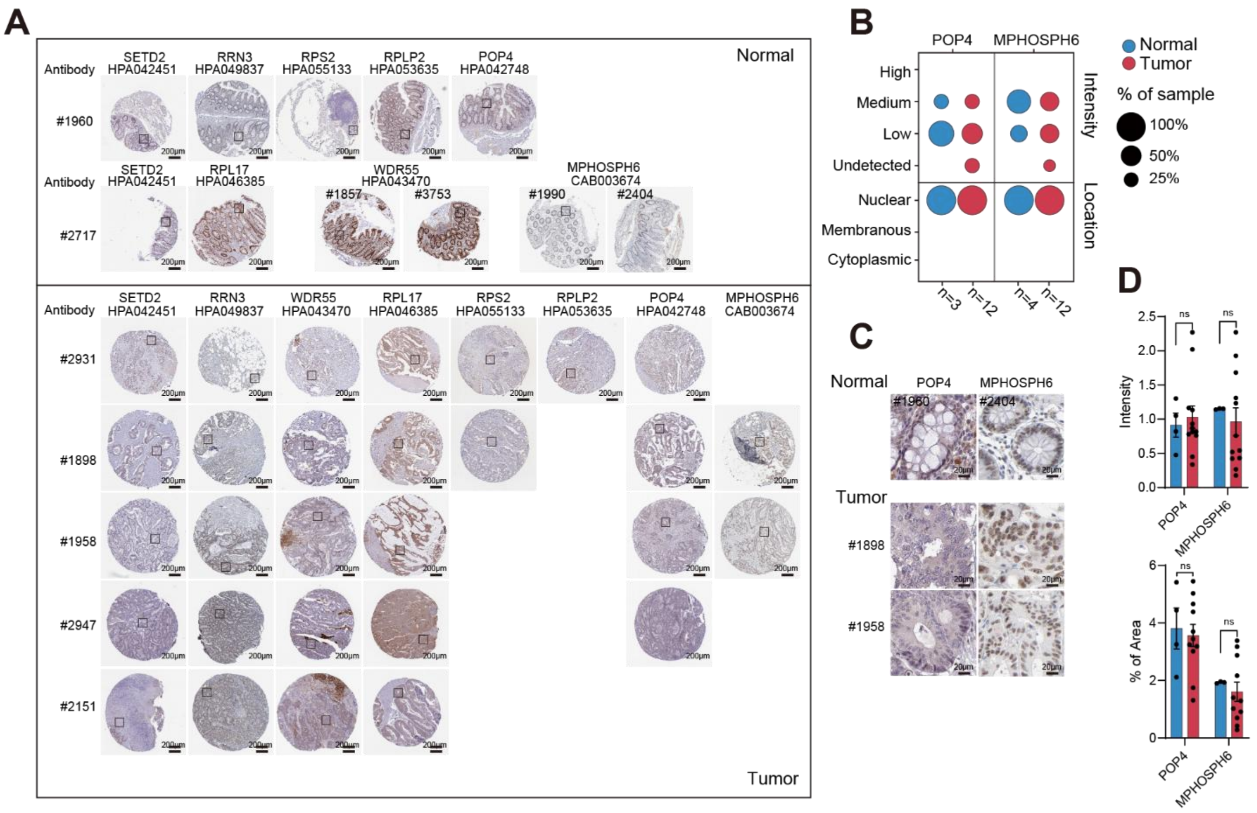

Figure S7. SETD2 loss is accompanied by disordered RBFs and RPs in human colorectal tumor.

(A) Representative immunohistochemistry images of colorectal tumors from the HPA. Scale bars, 200 μm. **(B)** Statistics on staining intensity and subcellular localization in immunohistochemical images derived from the HPA. The sample size for each group is indicated. **(C)** Representative immunohistochemistry images of colorectal tumors from the Human Protein Atlas. Scale bars, 20 μm. **(D)** Quantification of staining intensity and area percentage for the indicated proteins in immunohistochemical images derived from the HPA. Sample size for each group is detailed in Panel B. Statistical comparisons were made using a two-tailed Student's t test. Data are represented as mean ± SEM.
